# One dimensional transport in silicon nanowire junction-less field effect transistors

**DOI:** 10.1038/s41598-017-03138-5

**Published:** 2017-06-07

**Authors:** Muhammad M. Mirza, Felix J. Schupp, Jan A. Mol, Donald A. MacLaren, G. Andrew D. Briggs, Douglas J. Paul

**Affiliations:** 1 University of Glasgow, School of Engineering, Rankine Building, Oakfield Avenue, Glasgow, G12 8LT UK; 2 Department of Materials, University of Oxford, 16 Parks Road, Oxford, OX1 3PH UK; 3 University of Glasgow, SUPA School of Physics and Astronomy, Kelvin Building, University Avenue, Glasgow, G12 8QQ UK

## Abstract

Junction-less nanowire transistors are being investigated to solve short channel effects in future CMOS technology. Here we demonstrate 8 nm diameter silicon nanowire junction-less transistors with metallic doping densities which demonstrate clear 1D electronic transport characteristics. The 1D regime allows excellent gate modulation with near ideal subthreshold slopes, on- to off-current ratios above 10^8^ and high on-currents at room temperature. Universal conductance scaling as a function of voltage and temperature similar to previous reports of Luttinger liquids and Coulomb gap behaviour at low temperatures suggests that many body effects including electron-electron interactions are important in describing the electronic transport. This suggests that modelling of such nanowire devices will require 1D models which include many body interactions to accurately simulate the electronic transport to optimise the technology but also suggest that 1D effects could be used to enhance future transistor performance.

## Introduction

Complementary metal-oxide semiconductor (CMOS) transistor technology is the mainstay of electronics. The scaling of the technology to dimensions below ~45 nm where short channel effects start to become significant has required significant changes to the simple planar two-dimensional (2D) metal-oxide semiconductor field effect transistor (MOSFET)^1^. FinFET, double gate and/or fully-depleted approaches have been required for the 22 nm technology node to improve the electrostatic depletion of the transistor channel to improve the off-current, I_off_ whilst future scaling beyond the 10 nm technology node is predicted to require nanowire technology^2^ to improve the electrostatic control further^3^. Si nanowires have a multitude of potential applications beyond replacing CMOS transistors^2, 4^ which include semiconductor memories^5^, photovoltaics^6^, thermoelectric generators^7^, biosensors^8^, colour selective photodetectors^9^ and qubits^10^. Cryogenic CMOS is a recently developing area especially for quantum simulators or computing where it is clear that control electronics must operate at 4.2 K or less in dilution refrigerators to be able to control qubits^10^ and readout electronics^11^. The low temperature behaviour of transistors is therefore also becoming important for a few niche applications in research.

Most nanowire transistor designs to date rely on the formation of junctions between the heavily doped source and drain contacts and the undoped quasi-one-dimensional (1D) channel. Theoretical^12^ and experimental studies^13^ have shown that atomic-scale variations in the doping profile can lead to drastic variability in transistor behaviour. The need for ultrasharp source and drain junctions, however, imposes severe constraints on doping techniques and the processing thermal budget. Junction-less nanowire transistors^4^ do not suffer from these limitations, and can be fabricated without the need for ultrafast annealing techniques.

While the junction-less transistor was first envisioned by Lilienfield^14^ in 1925, working devices only became possible with the advent of silicon-on-insulator (SOI) technology. Junction-less transistors require high doping (ideally >1 × 10^19^ atoms cm^−3^) to ensure a high current drive and to minimize contact resistance. In 3D devices, however, such high doping makes it impossible to fully deplete the channel of charge carriers because of electrostatic screening of the gate electric field and so the highest currents cannot be realised. Screening is modified in 2D and 1D allowing the device channels to be fully depleted. For 1D nanowires only electrons external to the nanowire can screen the Coulomb potential^15^ which enables the complete pinch-off of the channel which is necessary to turn the device off even for doping concentrations well above the Mott metal-insulator transition^16^.

In this Letter we investigate the influence of dimensionality in electrostatic screening as well as in charge transport in the nanowire transistor and demonstrate a number of clear signatures of 1D electronic behaviour. In particular, we will focus on the universal conductance scaling behaviour for low bias voltages. Such universal conductance scaling has been previously observed in single walled carbon nanotubes^17^, Li_0.9_Mo_6_O_17_^18^ and metallic nanowires^19^, and is indicative for 1D charge transport. Low temperature transport also demonstrates clear Coulomb gap behaviour indicative of significant many body electron-electron interactions. Many simulations of junction-less transistors to date use 3D or 2D models^20–23^ to describe the electronic transport and to optimise the devices. We demonstrate that 1D transport models with many body interactions must be used to accurately describe the present devices when the nanowire diameters become less than the key electron transport length scales.

## Results

### Junction-less transistor behaviour

**Figure 1:**
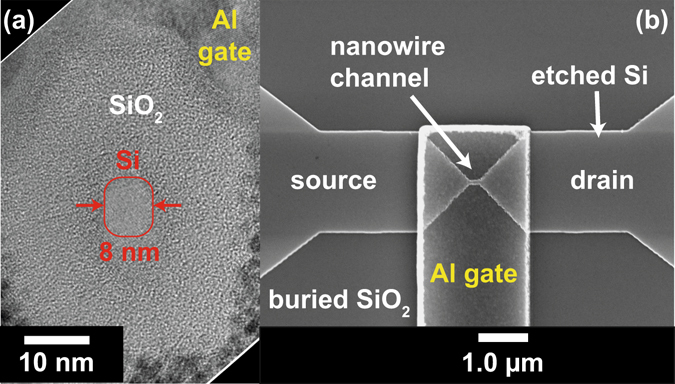
TEM and SEM images of the nanowire and transistor. (**a**) A cross sectional TEM image of the 8 ± 0.5 nm diameter nanowire with 16 nm SiO_2_ thickness. (**b**) A SEM image of the gate over the top of the Si channel and parts of the source-drain regions. The nanowire length is 150 nm.

**Figure 2:**
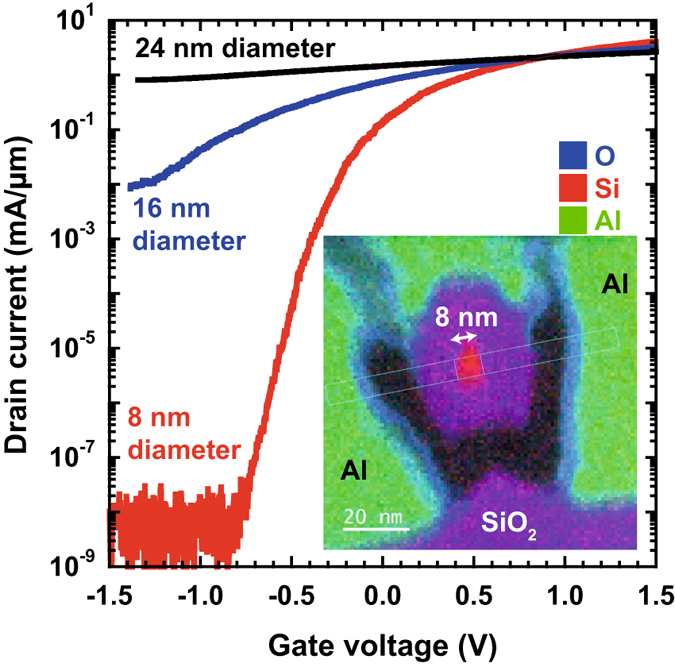
3D to 1D dimensional transport change with nanowire diameter. The drain current, *I*_*D*_ as a function of gate voltage for Si nanowires with three different diameters of 8 nm, 16 nm and 24 nm. The drain voltage, *V*_*D*_ = 1.5 V and all measurements are at 293 K. The insert is an elemental map of a cross-section of the smallest nanowire, measured by TEM-EELS, which was used to determine the nanowire diameter.

Figure 1 present a transmission electron microscope (TEM) image of the smallest nanowire with 8 ± 0.5 nm diameter along with a scanning electron microscope (SEM) lateral image demonstrating the gate overlapping the nanowire and the source and drain contact regions. Whilst the Al gate is much longer than the 150 nm long nanowire, the physical gate-length of the transistor will be determined by the nanowire length as the large source and drain sections are not depleted out by the gate (see Fig. 2). The nanowires for this work were implanted using P and previous measurements on sub-10 nm nanowires in Hall bar geometry devices at *T* = 1.4 K^5, 24^ have indicated an activated carrier density of 9.8 × 10^18^ cm^−3^. This is significantly above the Mott criterion of 3.5 × 10^18^ cm^−3^ indicating that the material is degenerately doped and should demonstrate metallic behaviour at all temperatures^16^.

First, we investigate the current–voltage behaviour of our junction-less transistors. We find that the devices with an 8 nm channel diameter behave nearly identically to a conventional MOSFET at 300 K, whereas the devices with larger channel diameters (16 ± 0.5 nm and 24 ± 0.5 nm) behave more like bulk, metallic conductors. Figure 2 demonstrates the drain current, *I*_*d*_, versus gate voltage, *V*_*g*_, for a drain voltage, *V*_*D*_ of + 1.5 V. The *I*_*off*_ is below the detection limit of the measurement setup (0.1 pA), and the on-current, *I*_*on*_ to *I*_*off*_ ratio between *V*_*g*_ = −0.5 V and *V*_*g*_ = +0.5 V is 1 × 10^8^, which clearly demonstrates the electrostatic depletion of charge carriers in the 8 nm diameter channel. By contrast, the same *I*_*on*_/*I*_*off*_ ratios for the 16 nm and 24 nm channels are only 10 and 2, respectively.

The subthreshold slope (SS), which is defined as the inverse of the slope of the log of the drain current versus gate voltage below threshold is 66 mV dec^−1^ for the 8 nm channel. The sharpness of the on-off switching ratio for these devices is close to the theoretical lower limit of SS = (*k*_B_ *T*/*q*)ln(10), which corresponds to 60 mV dec^−1^ at *T* = 300 K, and is nearly equal to the best SOI trigate transistors^25^ with SS values of 63 mV dec^−1^. For the larger diameter channels, the subthreshold slope increases to 570 mV/dec for the 16 nm diameter nanowire and >12000 mV/dec for the 24 nm diameter nanowire. All the nanowire devices for all diameters demonstrate high *I*_*on*_ values (all >10 *μ*A at 1.0 V). Setting the off-current, *I*_*off*_ at 100 nA/*μ*m gate width with a gate overdrive of 1.0 V produces an *I*_*on*_ of 1.15 mA/*μ*m which compares favourably to 0.61 mA/*μ*m measured previously from 25 nm gate-length Si MOSFETs^26^ and the 0.62 mA/*μ*m measured from 50 nm gate-length InGaAs MOSFETs^27^. The nanowire current has been divided by the diameter to achieve the current per unit gate width since as will be demonstrated later, the Fermi wavelength is significantly larger than the nanowire diameter indicating the electron wavefunctions completely fill the diameter of the nanowire. Higher voltages provide even higher performance: for example, *I*_off_ at 10 pA/*μ*m with a gate overdrive of 1.8 V, the present 150 nm gate-length nanowires have 2.52 mA/*μ*m drain current. This is significantly higher than the 0.92 mA/*μ*m from 80 nm gate-length high-voltage 3D tri-gate MOSFETs from a 22 nm system on chip commercial technology^25^.

## Discussion

The strong dependence of the current–voltage behaviour on the channel diameter suggests a dimensionality transition from 1D to 3D electronic transport behaviour between the 8 nm and 24 nm channels. Using a 3D carrier density *N*_3D_ = 9.8 × 10^18^ cm^−3^, we estimate the mean free path from elastic scattering as $\ell =2\pi \hslash {n}_{{\rm{1D}}}/{g}_{s}{g}_{v}q$ = 1.4 nm, where *n*_1D_ = *N*_3D_^1/3^. *g*_*s*_ = 2 and *g*_*v*_ = 2 are the spin- and valley-degeneracies. The Fermi wavelength *λ*_*F*_ = *g*_*s*_ *g*_*v*_/*n*_1D_ = 18.7 nm at 300 K, indicating that a transition takes place from diffusive 1D transport in the 8 nm diameter channels to diffusive 3D transport in the 24 nm nanowires. It is clear that the electrostatic screening of the Coulomb potential in 3D limits the ability of the gate in the widest nanowires to deplete the channel resulting in poor modulation (*I*_*on*_ to *I*_*off*_ ratio) and poor *I*_*off*_. In the 1D regime, as determined by the nanowire diameter becoming smaller than the *λ*_*F*_, the electrons in the 1D channel cannot screen the Coulomb potential from the gate and excellent gate modulation can be achieved. This is therefore the first piece of evidence suggesting that the electron transport in the 8 nm diameter nanowires could be 1D.

In clean, high-mobility 1D channels where $\ell$ is much longer than the gate-length, ballistic quantised current behaviour is expected^28, 29^. Clean channels can also demonstrate Luttinger liquid behaviour^17^ where electron and spin transport can be independent^30, 31^. The high, metallic doping levels in the present nanowires provide too much scattering to expect these types of 1D transport signatures and diffusive transport is to be expected from the spacing of the dopants and $\ell$. Dirty 1D transport signatures with diffusive transport dominating include universal conductance scaling^17, 19^, Coulomb gaps^32^, Coulomb blockade^33, 34^, zero bias anomalies^35^ or for very dirty materials hopping conduction^36^.

### Universal conductance scaling

**Figure 3:**
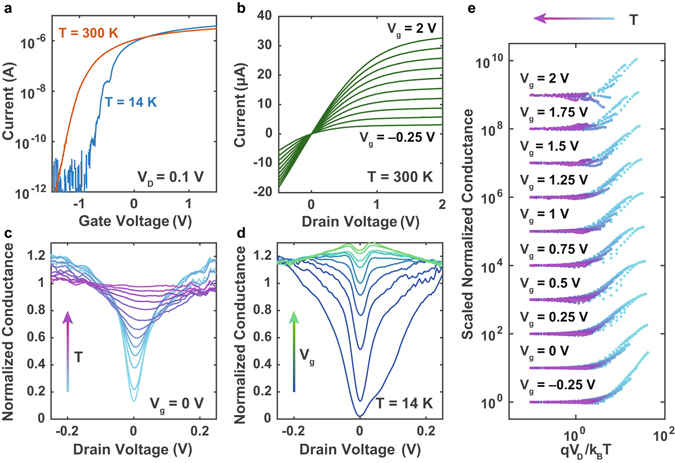
Universal conductance scaling. (**a**) The drain current versus gate voltage measured in the high-temperature (*T* = 300 K) and low-temperature (*T* = 14 K) regimes. In the low-temperature regime Coulomb blockade oscillations can be observed below the threshold voltage *V*_th_ ≈ − 0.25 V. (**b**) Room temperature current versus drain voltage (*I* − *V*_*b*_) traces are measured as a function of gate voltage. The *I* − *V*_*D*_ traces exhibit the typical transistor behaviour where the current *I* ∝ (*V*_*g*_ − *V*_th_)*V*_*D*_ − *V*_*D*_^2^/2 in the linear region (*V*_*D*_ < *V*_*g*_ − *V*_th_) and weakly dependent on *V*_*D*_ in the saturation region (*V*_*D*_ ≥ *V*_*g*_ − *V*_th_). (**c**) The normalized conductance (to 300 K) versus drain voltage as a function of temperature. There is a temperature dependent suppression of the conductance around zero bias which is superimposed onto the voltage dependent conductance. Normalizing the conductance by dividing it by the room temperature conductance results in a symmetric conductance gap centred around zero drain bias. (**d**) The normalized conductance versus drain bias voltage as a function of gate voltage. With increasing gate voltage, and carrier density, the conductance gap around zero drain bias gets softer, indicating enhanced screening of the Coulomb interaction between localized electrons. (**e**) A universal scaling plot of the conductance data, using a *T*-renormalized energy scale *qV*_*D*_/*k*_B_ *T* as described in Supplementary Information section I. All conductance traces for each gate voltage coincide, showing universal conductance scaling over a large gate range. The curves are scaled for each gate voltage and offset for clarity.

We will now discuss the universal conductance scaling observed in the 8 nm diameter nanowire transistors at low drain bias voltages, *V*_*D*_. Figure 3a demonstrates the source-drain current as a function of gate voltage measured at 300 K and at 14 K. We determine the threshold voltage *V*_th_ ≈−0.75 V from the room temperature *I* − *V*_*g*_ trace (see Supplementary Information) and *V*_th_ ≈−0.25 V from the 14 K trace. At 14 K the sub-threshold transport is dominated by single-electron transport^33, 37^, resulting in distinct Coulomb blockade peaks in the low-temperature *I* − *V*_*g*_ trace that are particularly pronounced below *V*_*g*_ = −1 V (Fig. 3a). The Coulomb oscillations in the sub-threshold transport are due to the transistor channel breaking up into one or more charge islands that are tunnel coupled to the source and drain electrodes^38^. The formation of these islands may either be due to disorder within the channel, e.g. surface roughness or random dopant fluctuations, or due to strain or confinement induced tunnel barriers at the extremities of the transistor channel. Previous studies of PADOX-fabricated silicon single-electron transistors^39^ have shown that strain may lead to tunnel barriers of at least 150 meV. Similarly, additional confinement potentials in constrictions, resulting from parasitic overexposure in the corners of the channel and the source/drain leads during the electron-beam lithography process, may give rise to tunnel barriers.

Above the threshold voltage, no Coulomb blockade oscillations are observed and the current at room temperature and 14 K approach the same value, indicating open-channel transport. It is in this open-channel transport regime that we will investigate universal conductance scaling. Figure 3b demonstrates *I* − *V*_*b*_ traces measured as a function of the gate voltage at 300 K. The *I* − *V*_*b*_ traces exhibit the characteristic linear behaviour for *V*_*D*_ < *V*_*g*_ − *V*_th_ and saturation behaviour for *V*_*D*_ ≥ *V*_*g*_ − *V*_th_. In the linear regime the current *I* ∝ (*V*_*g*_ − *V*_th_)*V*_*D*_ − *V*_*D*_^2^/2, and therefore the differential conductance *G* = *dI*/*dV* is proportional to −*V*_*D*_ for low bias voltages. Since any suppression of the differential conductance will thus be superimposed onto a linear dependence on *V*_*D*_, we normalize the differential conductance by dividing it by the room temperature conductance $\tilde{G}(V,T)=G(V,T)/G(V,T=300\,{\rm{K}})$. The normalized conductance as a function of drain bias voltage is presented in Fig. 3c for temperatures ranging from 14 K to 300 K, and in Fig. 3d for gate voltages ranging from −0.25 to 2 V. We estimate this approach to produce less than 10% uncertainty in the normalised conductance across the gap but larger uncertainty outside the gap.

At low temperatures there is a symmetric dip in the normalized conductance centred around zero drain bias, which reduces in depth and increases in width with increasing temperature (see Fig. 3c). Theoretical studies investigating the transition from ballistic to diffusive transport in quasi-1D conductors have attributed the occurrence of a zero-bias anomaly in these systems to electron-electron interactions which in dirty systems can lead to weak localization^35, 40, 41^ (we will demonstrate later that no weak localization is observed in the electron transport behaviour of the 8 nm diameter nanowires). These studies further predict that the characteristic energy of this Coulomb gap, determining both the width and the depth of the zero-bias dip, is inversely proportional to the number of channels *N* in the quantum wire. As we increase the gate voltage applied to our nanowire transistor (Fig. 3d), thereby increasing *N*, we observe a decrease in the conductance suppression around zero bias, in agreement with these predictions.

**Figure 4:**
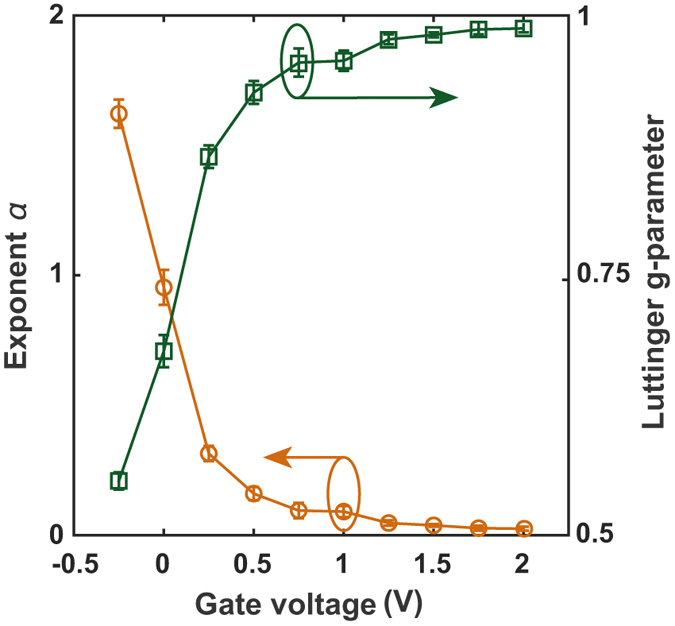
Voltage exponent for 1D transport. The measured voltage exponent *α* and Luttinger *g*-parameter extracted from the slope of the normalized conductance plotted on the *T*-renormalized loglog-scale.

The universal conductance scaling is illustrated in Fig. 3e, which demonstrates the scaled normalized conductance traces $\tilde{G}(V,T)/\tilde{G}(V\,\mathrm{=}\,0{\rm{V}},T)$ for each gate voltage plotted on a log-log scale as a function of the temperature-renormalized energy scale *qV*_*D*_/*k*_*B*_ *T*. To demonstrate that all traces measured for a particular gate voltage coincide, the vertical axis of all traces measured at each gate voltage have been scaled and offset by the same value (see Supplementary Information). The log-log plots in Fig. 3e demonstrate two distinct regions: (i) for $q{V}_{D}\ll {k}_{B}T$, where the conductance is constant; and (ii) for $q{V}_{D}\gg {k}_{B}T$, where the conductance follows a power-law dependence as a function of the *T*-renormalized energy scale. Similar universal scaling behaviour as shown in Fig. 3e has previously been observed in quasi-1D conductors, including multi-walled carbon nanotubes^17^, Li_0.9_Mo_6_O_17_^18^ and metallic nanowires^19^. From the slope of the log-log plots for $q{V}_{D}\gg {k}_{B}T$ we estimate the exponent *α* = 2/*g* − 2, where the Luttinger *g*-parameter^42^ defines the type of transport in 1D systems. For non-interacting fermions *g* = 1 and electronic transport should give Ohmic conductance^42^, i.e. the current or conductance should be linear with the voltage. Figure 4 presents the voltage exponent and Luttinger *g*-parameter as a function of gate voltage. For positive gate voltages *g* approaches 1 as the conductor becomes metallic. For negative gate voltages, *g* decreases as the carrier density decreases. The Luttinger parameter is given by Kane and Fischer^42^ for a single channel as

1 $$g\simeq \frac{1}{\sqrt{1+\frac{{q}^{2}}{2{L}_{e}{\varepsilon }_{0}{\varepsilon }_{r}{E}_{F}}}},$$

with the Fermi energy

2 $${E}_{F}=\frac{2{\pi }^{2}{\hslash }^{2}{n}_{1D}^{2}}{{g}_{s}^{2}{g}_{v}^{2}{m}^{\ast }},$$

where *m* ^*^ is the effective mass for the conduction band of silicon (0.19 *m*_0_ with *m*_0_ the free electron mass for conduction parallel to the (100) planes^43^) and *L*_*e*_ = *n*_1*D*_^−1^ is the spacing between the electrons. At a gate voltage −0.25 V we find *g* ≈ 0.55, hence we can estimate the carrier concentration from the above equations to be *n*_1*D*_ ≈ 6.7 × 10^6^ cm^−1^. If we use the *V*_*g*_ = −0.25 data from Fig. 3b at *V*_*D*_ = 1.5 V which was used to obtain *g* ≈ 0.55 with the low-field mobility, *μ* extracted from these channels^23^ of 107 cm^2^/Vs and consider the channel of radius *R* = 4 nm and length, *L* = 150 nm as a resistor then ${n}_{1D}={(\frac{{I}_{D}L}{q\mu \pi {R}^{2}{V}_{D}})}^{\frac{1}{3}}$. The extracted carrier density from this equation is 1.62 × 10^6^ cm^−1^. This is 4.1 times smaller than the value obtained from the single channel approach of Kane and Fischer^42^ suggesting that the nanowire may be a multi-channel conductor. Indeed 4 is the combined spin and valley degeneracy for the conduction band of silicon and if these degeneracies are added to the electron spacing by replacing *L*_*e*_ with *g*_*s*_ *g*_*v*_ *L*_*e*_ in equation 1 then the extracted density from the Kane model agrees with the experimental density. When the nanowire transistor is switched on, the saturation current regime is clearly Ohmic from the results in Fig. 4 with $g\sim 1$. As the transistor is switched off, the Luttinger parameters of *g* < 1 in the subthreshold region of Fig. 4 have been associated to 1D Luttinger liquid transport with long-range Coulomb interactions in carbon nanotubes^17^.

### Temperature dependence

**Figure 5:**
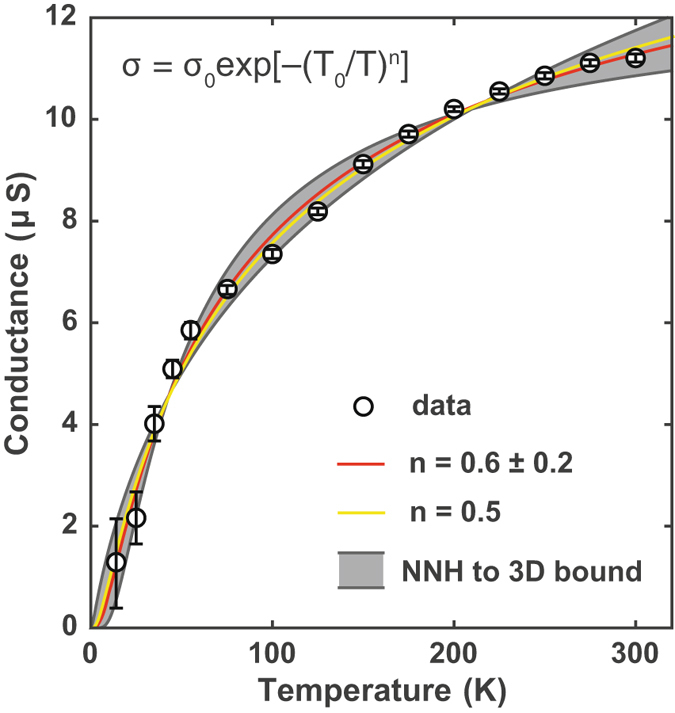
The temperature dependence for 1D transport The measured conductance at zero bias voltage and zero gate voltage as a function of temperature. The ‘best’ fit to the data (solid red line) gives a value *n* = 0.6 ± 0.2. A plot of the multi-channel Luttinger theory with *n* = 0.5 is also presented. The extracted transition temperature *T* = 13 ± 6 K is just below our measurement range, and does therefore not allow us to observe the onset of a Thouless crossover into the strong localization regime at low temperature. The grey shaded area indicates the bounds from excitation to the mobility edge or nearest neighbour hopping (NNH)^44^ to 3D variable range hoping^45^.

Finally, we investigate the temperature dependence of the zero-bias conductance measured for *V*_*g*_ = 0 V. At this gate voltage, the high-bias current does not have a Richardson like ${T}^{2}\exp (-\frac{W}{{k}_{B}T})$ dependence where *W* is the energy of the thermionic barrier height, allowing us to exclude any thermionic contributions to the zero-bias temperature dependence. Figure 5 demonstrates the zero-bias conductance measured as a function of temperature.

To understand Fig. 5, we need to consider a number of the length scales of the electron transport in the nanowire. As previously shown, *λ*_*F*_ ranges from 18.7 nm at 1.4 K to 20.4 nm at 300 K so the electron wavefunctions completely fill the whole of the nanowire providing 1D transport for the electrical current along the nanowire for all temperatures investigated. This therefore justifies using the diameter of the nanowire as the gate width when calculating the drain current per *μ*m of gatewidth.

The length scale for electron-electron interactions is the thermal length which results from the randomisation of the phase of an electron around the Fermi distribution from thermal smearing which produces an energy uncertainty of *k*_*B*_ *T* where *k*_*B*_ is Boltzmann’s constant. The thermal length is defined as ${L}_{T}=\sqrt{\frac{D\hslash }{{k}_{B}T}}$ where *D* is the diffusion constant ($=\frac{1}{2}{v}_{F}^{2}\tau$ with the Fermi velocity, *v*_*F*_ and the momentum relaxation time, *τ*). For the 8 nm Si nanowires and using the mobility extracted from *I*_*D*_ of 107 cm^2^/Vs and the extracted 1D density at *V*_*g*_ = −0.25 V of 1.62 × 10^6^ cm^−1^ ≡ 4.2 × 10^18^ cm^−3^, *L*_*T*_ is 32.5 nm at 1.4 K and decreases to 1.8 nm at 300 K. The cross over point from 1D electron-electron interactions to 3D corresponds to 17 K when *L*_*T*_ equals the nanowire diameter of 8 nm.

The best fit to the data in Fig. 5 produces

3 $$\sigma ={\sigma }_{0}\exp \,[-{(\frac{{T}_{0}}{T})}^{n}]$$

with a temperature exponent of *n* = 0.6 ± 0.2 and the extracted transition temperature, *T*_0_ = 13 ± 6 K. There are no theories with *n* = 0.6 but there are a number of theories with *n* = 0.5 which is within the experimental uncertainty^32, 35, 36, 46^. Weak localisation due to the Altshuler and Aronov corrections^46^ produce a ${T}^{-\frac{1}{2}}$ temperature dependence in both 1D and 3D which clearly does not fit the data. In the variable range hopping transport regime, the exponent is $n=\frac{1}{1+d}$ where *d* is the dimension. For 1D variable range hopping the exponent is therefore $n=\frac{1}{2}$ but since the transition temperature is given by ${T}_{0}=\frac{4}{{a}_{B}^{\ast }{k}_{B}g({E}_{F})}$ where ${a}_{B}^{\ast }=2.3\,{\rm{nm}}$ is the effective Bohr radius and *g*(*E*_*F*_) = 5.7 × 10^28^ is the density of states at the Fermi level, *E*_*F*_ = 16.2 meV this produces *T*_0_ = 2160 K and clearly does not fit the data. Since the zero-bias anomaly suggests a Coulomb gap in the density of states, variable range hopping with a Coulomb gap also has a temperature exponent of ${T}^{-\frac{1}{2}}$ universally for all dimensions^32, 36^. The theoretical value of ${T}_{0}=\frac{2.8{q}^{2}}{4\pi {\varepsilon }_{0}{\varepsilon }_{0}{a}_{B}^{\ast }{k}_{B}}$ = 1679 K, however, and so again this model^36^ does not fit the data either.

A clean, single channel Luttinger liquid is predicted to have a *T* ^*α*^ where *α* is related to the the many body interactions in the system^17^. Whilst this clean Luttinger liquid model does not fit the present nanowire data, Mischenko *et al*.^35^ have developed a multi-channel Luttinger liquid model for disordered nanowires which produces a temperature behaviour for the electrical conductivity identical to equation 3 with *n* = 0.5 temperature exponent and the model does fit the present data within experimental uncertainty and produces the number of channels to be 3.9 ± 0.9 so approximately 4. This model has been plotted in Fig. 5 as the *n* = 0.5 model and it is the only model that fits the data and produces fit parameters in agreement with the theoretical predictions of any of the tested models. As the conduction band of silicon has a spin degeneracy of 2 and a valley degeneracy of 2, it would suggest that the 4 channels in the silicon nanowire are just related to the spin and valley degeneracy. Whilst separation of spin and charge transport is the unique signature of Luttinger liquid behaviour in 1D nanowire systems^30, 31^, which is beyond our present experiments, the disordered multi-channel Luttinger liquid model provides the best fit to the present data. It has been suggested that silicon nanowires with widths around 3 nm will have the valley degeneracy lifted^47^. Therefore the scaling to smaller nanowire widths should result in changes to both *g* and *α* which would provide further proof of the Luttinger liquid behaviour but this is beyond the scope of the present work.

To conclude, clear 1D electron transport consistent with the experimentally determined Fermi wavelength, thermal length, zero-bias anomaly and a disordered multi-channel Luttinger liquid model behaviour are observed in the 8 nm diameter Si nanowires at low temperatures. Observation of universal scaling curves and an exponential temperature dependent electrical conductance with a $n=\frac{1}{2}$ exponent also provide strong evidence for 1D behaviour up to room temperature. The Fermi wavelength indicates that the wavefunctions completely fill the diameter of the nanowire for all temperatures tested demonstrating quasi-1D transport even at room temperature and the electron-electron interactions in the nanowire are 1D for temperatures below 17 K. These results indicate that accurate modelling of such nanowires for future CMOS or quantum electronics will require 1D models which include many body interactions as the experimentally observed electron transport cannot be explained by the presently used 3D theories. This also opens up the possibility of deliberately using 1D effects to enhance transistor performance in future CMOS technologies.

## Methods

### Fabrication

The devices were fabricated from 55 nm SOI wafers from SOITEC with a 145 nm buried oxide which were implanted with P at 15 keV with a dose of 4 × 10^14^ cm^−2^ before being annealled at 950 °C for 90 seconds. The top Si was then etched to reduce the thickness for the smallest dimension nanowires before a Vistec VB6 electron beam lithography tool was used to pattern the nanowire using hydrogen silsesquioxane (HSQ) resist. A low damage SF_6_/C_4_F_8_ inductivity coupled plasma etch was undertaken^48^ before the resist was stripped and a thermal oxide was grown at 950 °C. Optical lithography was then used to define electrical contacts using 20 nm of Ni and 50 nm of Pt after the oxide had been stripped with HF. An anneal in forming gas at 380 °C for 15 minutes was used to alloy the contacts forming a NiSi Ohmic contact with a specific contact resistance of 0.8 Ω-mm. Finally, electron beam lithography was used with 400 nm of PMMA resist to lift-off the Al gate.

### Measurement

The dc current-voltage characteristics for Fig. 2 were measured using an Agilent B1500 semiconductor parameter analyser at room temperature (293 K) with a Cascade Microtech probe station with a noise floor of ≈0.1 pA and *V*_*D*_ = 1.5 V. The data for Figs 3, 4 and 5 were also obtained by dc techniques using the Agilent B1500 but with a Lakeshore CRX-6.5K Cryogenic Probe station with a higher noise floor of ≈1 pA. The original electrical dataset from the nanowires analysed in this paper are avalable at http://eprints.gla.ac.uk/140323/7/140323Suppl.xlsx.

### TEM analysis

Samples were prepared for TEM analysis using standard ‘lift-out’ procedures on a FEI Nova Dualbeam Focused Ion Beam system. TEM and STEM were conducted on a JEOL ARM200cF instrument equipped with a cold field emission gun that was operated at 200 kV and a CEOS (probe) aberration corrector. EELS data were collected using a Gatan 965 Quantum ER spectrometer using the Dual EELS^49^ and Spectrum Imaging^50^ methodologies. Energy dispersive x-ray spectroscopy (EDS) was conducted simultaneously using a Bruker XFlash detector.

## Electronic supplementary material

Supplementary Information
