# Supplementary material for: One dimensional transport in silicon nanowire junction-less field effect transistors
Source: Sci Rep. 2017 Jun 7;7:3004. doi: 10.1038/s41598-017-03138-5 (PMC5462787; doi:10.1038/s41598-017-03138-5)
Supplement: Supplementary file 1 — Supplementary Information [file 41598_2017_3138_MOESM1_ESM.pdf]

One dimensional transport in silicon nanowire field effect transistors: supplementary information

Muhammad M. Mirza,[1] Felix J. Schupp,[2] Jan A. Mol,[2] Donald A. MacLaren,[3] G. Andrew D. Briggs,[2] and Douglas J. Paul[1, a)]

[1)] *University of Glasgow, School of Engineering, Rankine Building, Oakfield Avenue, Glasgow, G12 8LT, United Kingdom*

[2)] *Department of Materials, University of Oxford, 16 Parks Road, Oxford OX1 3PH, UK*

[3)] *University of Glasgow, SUPA School of Physics and Astronomy, Kelvin Building, University Avenue, Glasgow, G12 8QQ, United Kingdom*

PACS numbers: 73.63.-b, 62.23.Hj, 73.22.-f

Keywords: silicon nanowire, electronic transport, 1D

[a)] Electronic mail: Douglas.Paul@glasgow.ac.uk

I. FITS FOR THE EXPONENT α

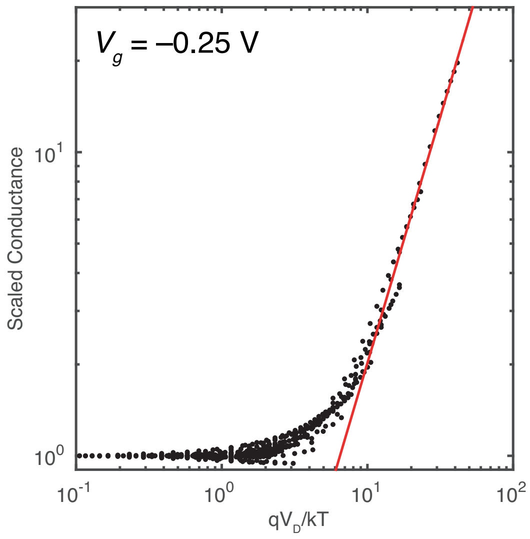

FIG. 1. Scaled conductance as a function of bias energy normalized by thermal energy for all temperatures taken at -0.25 V gate voltage.

The values plotted in Fig. 4 of the main paper, were extracted from the fits in Fig. S1 and Fig. S2, that show the scaled conductance as a function of bias energy normalized by thermal energy for all temperatures at different gate voltages.

The scaled normalized conductance traces that were offset for clarity in Fig. 3 of the main paper were calculated using:

$$10^{j-1}\left[10\frac{\tilde{G}_j(V_b,T)/[\tilde{G}_j(0,T)-1]}{\tilde{G}_j(V_{b0},T)/[\tilde{G}_j(0,T)-1]}+1\right],$$

where $\tilde{G}_j(V_b,T)$ denotes $G(V_b,T)/G(V_b,300)$ of the jth gate voltage.

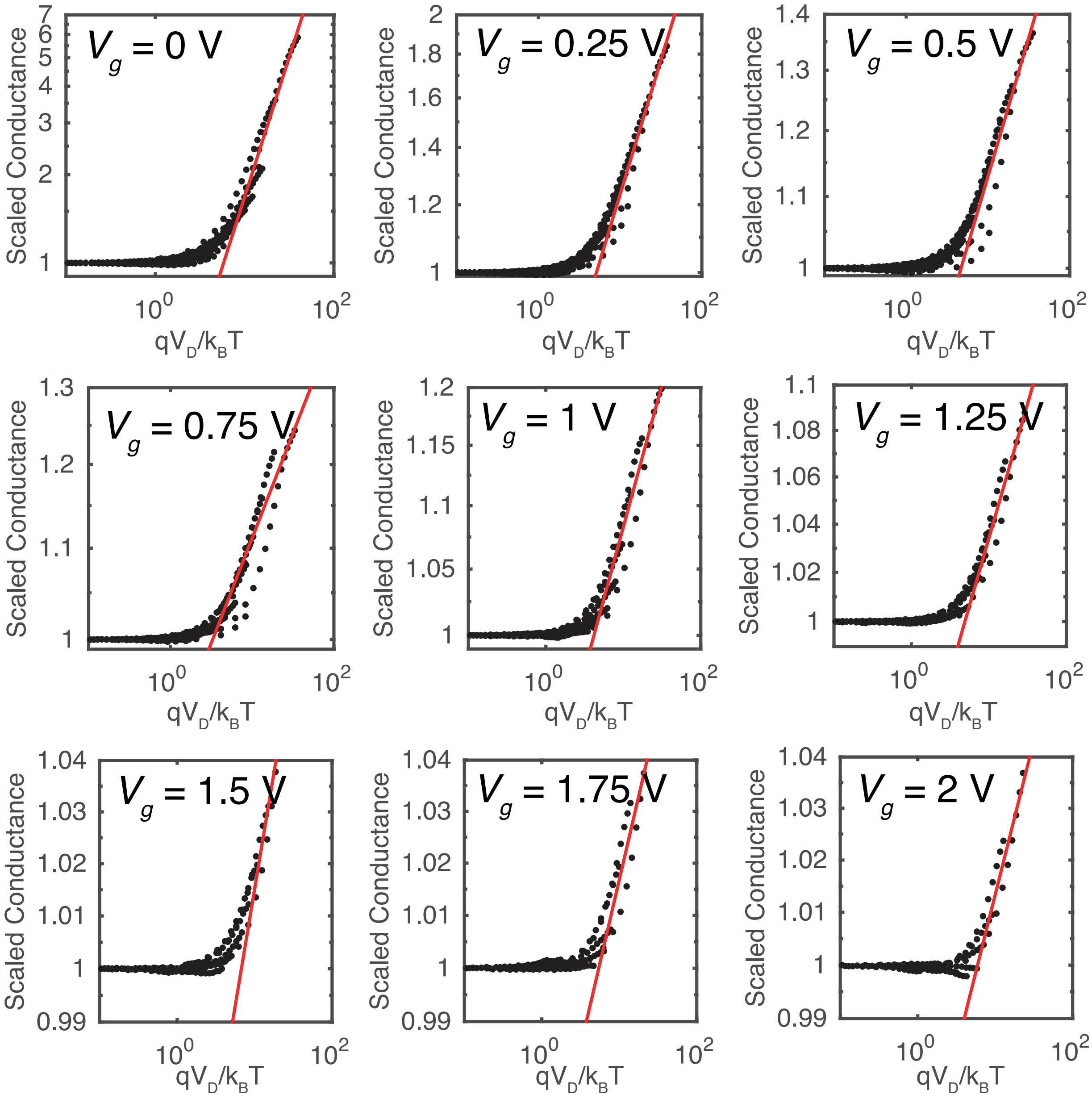

FIG. 2. Scaled conductance as a function of bias energy normalized by thermal energy for all temperatures taken at different gate voltages.

II. CALCULATION OF THE NUMBER OF CHANNELS ACCORDING TO DISORDERED MULTI-CHANNEL LUTTINGER LIQUID THEORY

Following Mishchenko's theory[1] the conductance at zero bias as a function of temperature should behave according to the following expression:

$$G(T) \sim \exp\left(-1.07\sqrt{\frac{T^*}{T}}\right) \tag{1}$$

Using this expression we can fit the data in Fig. 5 from the main paper in order to find $T^* = 94 \pm 14$ K. From that we can estimate the number of channels involved in the transport, N according to the following expressions from Mishchenko's theory[1]:

$$N = \frac{p_i \hbar}{\pi \tau T^* k_B} \tag{2}$$

where N is the number of channels involved in the transport, k_B is Boltzmann's constant and τ is the elastic momentum relaxation time. The mobility in the present nanowires has been measured[2] in the low-field drift regime as 107 $\mathrm{cm}^2/\mathrm{Vs}$ and using

$$\tau = \frac{q}{m^*} \tag{3}$$

where q is the electron charge, the effective mass $m^* = 0.19m_0$ [Ref. 3] and therefore $\tau = 11.4$ fs is the elastic momentum relaxation time. p_i is defined by the following expression:

$$p_i = \frac{q^2}{16\epsilon_0\epsilon_r\hbar v_F}\log\left(d/R\right) \tag{4}$$

where $d = 26 \pm 0.5$ nm is the radial distance to the gate, $R = 4 \pm 0.5$ nm is the nanowire radius, ϵ_0 is the vacuum- and $\epsilon_r = 3.9$ the relative permittivity of silicon dioxide.

Next we need to estimate the Fermi-velocity using the charge carrier density in one dimension $n_{1D} = 1.62 \times 10^6\ \mathrm{cm}^{-1}$ as extracted from the measurements to produce,

$$v_F = \frac{\hbar 2\pi n_{1D}}{g_s g_v m^*} = 6.38 \times 10^6 \mathrm{ms}^{-1} \tag{5}$$

where $g_s = 2$ is the spin degeneracy, $g_v = 2$ is the valley degeneracy. From this calculation we get the number of occupied channels $N = 3.9 \pm 0.9$.

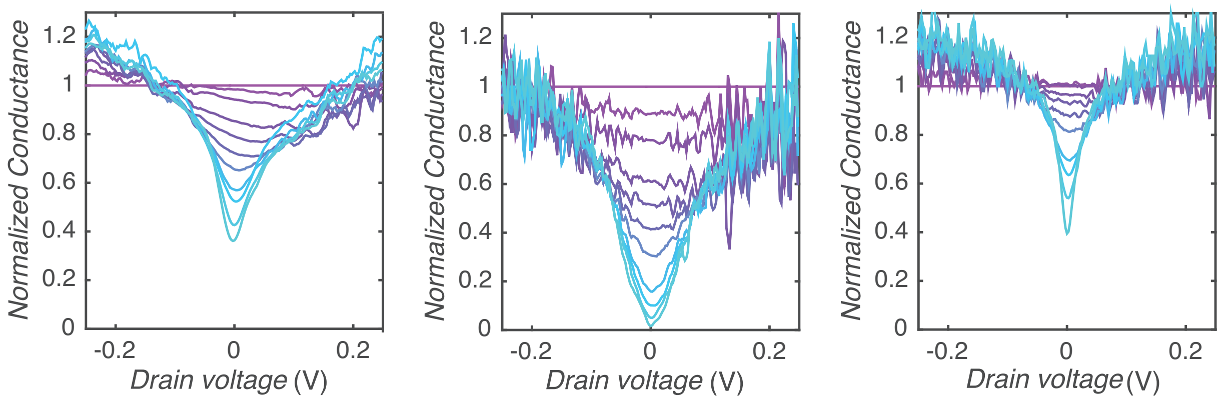

FIG. 3. Normalized conductance as a function of drain voltage at temperatures from 14 K to 300 K in 3 other devices with the same dimensions (8 nm diameter by 150 nm length nanowire) and doping density as the device from the main text.

III. OTHER SAMPLES

This section presents data taken in measurements on other nominally identical devices fabricated in the same batch as the device analysed in the main text to demonstrate that the reported 1D electronic transport results are reproducible across many devices. Fig. 3 demonstrates the normalized conductance in these devices as a function of drain voltage for different temperatures from 14 K to 300 K. Identical to the device in the main text, we observe the characteristic zero bias feature, that disappears with increasing temperature.

IV. UNCERTAINTIES

The uncertainty in the width of the nanowires was obtained by a number of line scans taken on electron energy loss spectroscopy (EELS) data taken across different diameters of the nanowires. The line scans for both the Si content and O content were used as both demonstrate the interface between the Si nanowire and the SiO2 gate oxide. The measurements from 4 separate line scans at different angles produced an uncertainty of ± 0.5 nm.

The error bars in both Figures 4 and 5 were obtained from the 90% confidence bounds of a polynomial fit of the current around the zero bias voltage. A 90% confidence level was used due to the estimated 10% uncertainty in obtaining the normalized conductance for the

zero-bias gap in Figure 3.

REFERENCES

[1]Mishchenko, E. G., Andreev, A. V. & Glazman, L. I. Zero-Bias Anomaly in Disordered Wires. *Phys. Rev. Lett.* **87**, 246801 (2001).

[2]Georgiev, V. P. *et al.* Experimental and simulation study of 1D silicon nanowire transistors using heavily doped channels (2017). *IEEE Trans. Nanotechnol.* (Accepted for Publication).

[3]Stern, F. & Howard, W. E. Properties of Semiconductor Surface Inversion Layers in the Electric Quantum Limit. *Phys. Rev.* **163**, 816–835 (1967).
